# Acute Renal Failure after Consumption of Fish Gall Bladder

**DOI:** 10.1155/2014/194129

**Published:** 2014-02-02

**Authors:** Nishant Raj Pandey, Bian Yu Yao, Sudha Khakurel

**Affiliations:** ^1^Grande International Hospital, Kathmandu, Nepal; ^2^China National Petroleum Corporation Central Hospital, China; ^3^Tianjin Medical University General Hospital, Tianjin, China

## Abstract

A case of acute renal failure after consumption of fish gall bladder as traditional medical remedy is reported. The patient fully recovered with conservative treatment. The risk of acute kidney failure and even multiple organ dysfunction syndrome following ingestion of fish gall bladder is highlighted.

## 1. Introduction

In India, especially in Assam, people believe that fish gall bladder can improve vision and treat rheumatism. Chinese populations have a similar view and believe it improves eyesight and cure asthma [1]. Due to frequent consumption of fish gall bladder, fish bile poisoning cases care reported more commonly in China, India, Japan, and other Asian countries [2–4]. There were many reports about fish gall bladder poisoning leading to acute renal failure (ARF), acute liver injury, and therefore increasing mortality [5]. The incidence of ARF in fish bile poisoning is 55%–100%, while the mortality rate accounts 91.7%. Recently, studies have shown that fish gall bladder can also damage the heart, liver, and gastrointestinal tract and lead to multiple organ dysfunction syndrome (MODS) [1]. This is a case of gall bladder poisoning leading to acute renal failure in Chinese patient. This report is a whole new perspective on the pathogenesis of acute renal failure on complements about cases of poisoning in fish guts. This has a positive role in guiding treatment of fish bile poisoning, with obvious effect to improve its prognosis.

## 2. Case Features

A 56-years-old Chinese woman with past medical history of chronic bronchitis presented to a community hospital after consumption of grass carp fish gall bladder. Fish gall bladder was about 2 × 2 cm. Initial symptoms were nausea, vomiting, abdominal cramps, and watery diarrhea. After the onset of the symptoms she was treated in community level hospital with intravenous infusion for three days which did not improve her condition. After 5 days, she was presented in Tianjin Medical University General Hospital, Emergency Medical Center, for better management. On examination vital signs were stable. However, oliguria or anuria was observed in 24 hours and showed no edema.

Blood works revealed the following: WBC: 7.82 × 10^9^/L, N: 80.91%, and HB: 168 g/L; ABG revealed the following: PH: 7.50, PO^2^: 153 mmHg, Lac: 1.4 mmol/L, HCO^3−^: 25.7 mmol/L, and BE: 2.7 mmol/L. Urinanalysis reported: RBC: 0/HPF, WBC: 0/HPF, pathological casts: 0/LPF, specific gravity: <1.005, PH: 6.3; biochemistry reported the following: Cr: 277 umol/L, BUN: 13.31 mmol/L, ALT: 95 U/L, AST: 35 U/L, and TBIL: 85.9 mmol/L.

After admission intravenously reduced glutathione 1.2 grams was administrated once a day for live protection and injection of 9AA compound amino acid 250 mL was administrated once a day as supplement amino acids and intravenous pantoprazole 40 mg was administrated twice a day. Oral sodium bicarbonate tablets 1 g were given thrice a day as it promotes fish bile toxins from the urethra, oral lactulose 10 mL was given thrice a day to promote fish bile toxins from the intestinal tract; and traditional Chinese medicine (Niaoduqing) was also given to protect renal function. After admission hemodialysis was performed three times. On second day of admission urine output increased to 1800ml and biochemistry reviewed Cr: 303 umol/L, BUN: 19.2 mmol/L, Na: 127 mmol/L, CL: 88 mmol/L, and AG: 23.73. After the 11th day of admission, urine volume reaches 7600 mL/24 h, On arterial blood gas (ABG) Examination: PH: 7.41, PO_2_: 90 mmHg, Lac: 0.7 mmol/L; HCO^3−^: 21.6 mmol/L; BE: −2.6 mmol/L; On Urine examination: RBC: 7/HPF, WBC: 6/HPF, pathological tube: 0/LPF, gravity: <1.005, PH: 6.3; On stool routine examination: occult blood negative.

Considering ARF adequate rehydration and electrolyte balance was maintained. While continuing the above drugs, 1000 mL of 5% Dextrose and sodium chloride injection and 0.9% sodium chloride injection 500 mL were given for adequate rehydration, and intravenous 10% potassium chloride 15 mL and oral salt capsules were administrated to maintain rehydration and electrolyte balance. However, patient still complained of recurrent vomiting and nausea. Therefore, fibrotic endoscopy was planned which showed chronic gastritis and duodenal inflammation, and systematic treatment was also given.

After a month of admission, urine volume was 2000 mL/24 h, Cr: 124 umol/L, BUN: 7.1 mmol/L; ALT: 45 U/L, AST: 30 U/L, GGT45 U/L; Patients started normal diet. Her vomiting symptoms improvement and was then discharged.

## 3. Discussions

Most of the fish poising contains ciguatoxin [6] and mackerel poison, which are common in marine coral fish. Grass carp is usually without the toxin and does not have the characteristics of perishable mackerel [7]. However, grass carp bile contains highly virulent bile toxins, which cannot be damaged easily by ethanol or heat. One of the main toxic components is water soluble sodium cyprinol sulphate, which can lead to multiple organ dysfunctions [5]. Renal failure is the most commonly reported effects of fish bile poisoning [8]. It is believed that the fish bile results into serious damage to renal tubules. Deng et al. [9] found that light microscopy showed damages to epithelial cells in the proximal tubule and focal destruction of epithelial cells. Electron microscopy showed that mitochondria crista of epithelial cells in the proximal tubules had decreased or disappeared and the renal mesangium was extended. Glomerular cells were swollen and podocytes were partially fused; lysosomes were broken. Partial potocytic processes are fused. It is believed that the toxin in fish gall bladder damages or breaks lysosomes, meanwhile inhibiting cytochrome oxidase and blocking cellular energy metabolism, so as to cause necrosis of the proximal tubular epithelial cells. From our case, we can hypotise that due to frequent vomiting, diarrhea and insufficient intake there was significant decreased blood volume, leading to kidney blood stasis which worsen renal failure. Therefore, for these patients on dialysis, active rehydration should have significant role in prognosis.

Recently, studies have shown that fish gall bladder can also damage the heart, liver, and gastrointestinal tract and lead to multiple organ dysfunction syndrome (MODS) in addition to ARF [9]. The effect of fish bile in human body mechanism needs further study and physicians should have more attention in management of fish bile poising for better prognosis.
